# The association between preoperative serum cholinesterase and all-cause mortality in geriatric patients with hip fractures: a cohort study of 2387 patients

**DOI:** 10.1186/s13741-024-00443-2

**Published:** 2024-07-24

**Authors:** Yan-Ning Zhang, Peng Xiao, Bin-Fei Zhang

**Affiliations:** 1 Department of Joint Surgery, Honghui Hospital, Xi’an Jiaotong University, No. 555 Youyi East Road, Beilin District, Xi’an, Shaanxi Province 710054 China; 2 Department of Orthopedics (International Ward), Hospital of Chengdu University of Traditional Chinese Medicine, Chengdu, Sichuan Province China

**Keywords:** Cholinesterase, Mortality, Elderly patients, Hip fracture, Regression

## Abstract

**Objective:**

This study is to evaluate the association between preoperative cholinesterase levels and all-cause mortality in geriatric hip fractures.

**Methods:**

Elderly patients with hip fractures were screened between Jan 2015 and Sep 2019. Demographic and clinical characteristics of patients were collected. Linear and nonlinear multivariate Cox regression models were used to identify the association between preoperative cholinesterase levels and mortality in these patients. Analyses were performed using EmpowerStats and the R software.

**Results:**

Two thousand three hundred eighty-seven patients were included in this study. The mean follow-up period was 37.64 months. Seven hundred eighty-seven (33.0%) patients died due to all-cause mortality. Preoperative cholinesterase levels were 5910 ± 1700 U/L. Linear multivariate Cox regression models showed that preoperative cholinesterase level was associated with mortality (HR = 0.83, 95% CI: 0.78–0.88), *P* < 0.0001) for every 1000 U/L. However, the linear association was unstable, and nonlinearity was identified. A cholinesterase concentration of 5940 U/L was an inflection point. When preoperative cholinesterase level < 5940 U/L, the mortality decreased by 28% for every 1000 U/L increase in cholinesterase (HR = 0.72, 95%CI: 0.66–0.79, *P* < 0.0001). When cholinesterase was > 5940 U/L, the mortality was no longer decreased with the rise of cholinesterase (HR = 1.01, 95%CI: 0.91–1.11, *P* = 0.9157). We found the nonlinear association was very stable in the propensity score-matching sensitive analysis.

**Conclusions:**

Preoperative cholinesterase levels were nonlinearly associated with mortality in elderly hip fractures, and cholinesterase was a risk indicator of all-cause mortality.

**Trial registration:**

This study is registered on the website of the Chinese Clinical Trial Registry (ChiCTR: ChiCTR2200057323) (08/03/2022).

## Introduction

The prevalence of hip fracture in elderly people increases with the global population growth, and is expected to become 2.6 million by 2025 and 7.3–21.3 million by 2050 (Gullberg et al. 1997; Sambrook and Cooper 2006). Downey et al. have reported a mean overall one-year mortality rate of 22% for hip fractures (Downey et al. 2019). Surgeons have attempted to use various treatment strategies to decrease mortality and improve prognosis, such as accelerated surgery (Investigators 2020), general anesthesia (Neuman et al. 2021), nutritional intake (Rizzoli et al. 2021), and operative choices (Investigators et al. 2019). However, these strategies do not improve prognosis. Many factors affect pathophysiology and play a negative role in geriatric hip fractures.

Cholinesterases are easily overlooked indicators. This enzyme family contains two members: acetylcholinesterase and butyrylcholinesterase. The primary biological purpose of the former is to regulate acetylcholine, a neurotransmitter, via hydrolysis at neuromuscular junctions, thus proving itself to be an essential component in the maintenance and performance of nervous systems (Taylor et al. 2009). Butyrylcholinesterase, previously called “pseudocholinesterase,” is produced in the liver (John et al. 2009) and is known as serum cholinesterase. Accumulated evidence suggests that serum cholinesterase activity is an indicator of liver function in patients with liver disease (Meng et al. 2013; Kaufman 1954; Tan et al. 2019). Moreover, serum cholinesterase is closely associated with the synthesis of albumin in the liver (Levine and Hoyt 1950) and is a well-known marker of liver dysfunction. In addition, serum cholinesterase concentration has been used to evaluate nutritional status in daily practice (Santarpia et al. 2013) and is commonly elevated in fatty liver and diabetes (Nomura et al. 1986; Inacio Lunkes et al. 2006). Furthermore, it has been reported that serum cholinesterase level is associated with in-hospital mortality in elderly patients with acute ischemic stroke (Li et al. 2020) and severe COVID-19 pneumonia and mortality (Nakajima et al. 2021). Regarding cholinesterase intervention, its inhibitors may reduce all-cause mortality and the risk of a second hip fracture throughout the first year after surgery (Tamimi et al. 2017; Eimar et al. 2013).

However, the relationship between serum cholinesterase levels and prognosis of patients with hip fractures remains unclear. Therefore, the present study assessed the influence of serum cholinesterase levels on patient mortality over a long-term follow-up period. We hypothesized that there would be either a linear or nonlinear association between preoperative cholinesterase levels and mortality. In this cohort study, we aimed to identify the role of cholinesterase levels on hip fractures.

## Methods

### Study design and setting

The work has been reported in line with the STROCSS criteria (Agha et al. 2019). All methods involving human participants were carried out in accordance with the 1964 Declaration of Helsinki and its later amendments. The Ethics Committee of Honghui Hospital approved this study, Xi’an Jiaotong University (No. 202201009, approval on Jan 28th, 2022). The follow-up data was obtained from all subjects and/or their legal guardian(s) by telephone and was approved by the Ethics Committee of Honghui Hospital, Xi’an Jiaotong University.

### Participants

Demographic and clinical data of patients were obtained from original medical records. Inclusion criteria were as follows: (1) age ≥ 65 years old; (2) a radiograph or computed tomography diagnosis of a femoral neck, intertrochanteric, or subtrochanteric fracture; (3) patients who were receiving surgical or conservative treatment in a hospital; (4) availability of clinical data when in the hospital; and (5) patients able to be contacted by telephone. Patients who could not be contacted were excluded from this study (Liu et al. 2022).

### Hospital treatment

Patients were examined using blood tests and ultrasonography to prepare for surgery. Intertrochanteric fractures are often managed with closed/open reduction and internal fixation (ORIF) of proximal femur nail anti-rotation. Femoral neck fractures are often treated with hemiarthroplasty (HA) or total hip arthroplasty (THA) (Liu et al. 2022).

### Follow-up

After discharge, patients’ family members were contacted by telephone from Jan 2022 to Mar 2022 to record survival data. Two medical professionals conducted this follow-up.

### Variables

Variables in our study were as follows: age, sex, occupation, history of allergy, injury mechanism, fracture classification, presence of hypertension, diabetes, coronary heart disease (CHD), arrhythmia, hemorrhagic stroke, ischemic stroke, cancer, associated injuries, dementia, chronic obstructive pulmonary disease (COPD), hepatitis and gastritis, age-adjusted Charlson comorbidity index (aCCI), time from injury to admission, time from admission to operation, preoperative cholinesterase level, operation time, blood loss, infusion, transfusion, treatment, stay in hospital, and follow-up. Occupations included those retired, farmers, and others. Injury mechanisms included falls, accidents, and others. The aCCI was a correction variable of the final score by adding 1 point for every decade over 40 years of age. Therefore, it is especially suitable for geriatric patient populations (Dan-Long et al. 2023).

Preoperative cholinesterase level was defined by the examination in the blood test at admission. The dependent variable was all-cause mortality, while the independent variable was preoperative cholinesterase level. Other variables were potentially confounding factors.

### Statistics analysis

Continuous variables are reported as mean ± standard deviation (Gaussian distribution) or median (range, skewed distribution). Categorical variables are indicated as numbers with proportions. Chi-square (categorical variables), one-way analysis of variance (ANOVA [normal distribution]), or Kruskal-Wallis H test (skewed distribution) were used to detect differences between different preoperative cholinesterase levels. Univariate and multivariate Cox proportional hazards regression models were used to test the association between preoperative cholinesterase levels and mortality. To test the robustness of our results, we examined the possibility of nonlinearity using a Cox proportional hazards regression model with cubic spline functions and smooth curve fitting (penalized spline method).

In addition, propensity score matching (PSM) was introduced for comparison between matched groups, and we adjusted for confounding factors in PSM models (Liu et al. 2022). Hazard ratios (HR) and 95%CI were calculated. *P* > 0.05 (two-sided) was considered statistically significance.

All analyses were performed using statistical software packages R (http://www.R-project.org, R Foundation) and EmpowerStats (http://www.empowerstats.com, X&Y Solutions Inc., Boston, MA, USA).

## Results

### Patient characteristics

From the initial 2887 participants who had hip fractures between Jan 2015 and Sep 2019, 2387 participants met the study criteria and were enrolled in our study. The mean follow-up period was 37.64 months. Seven hundred eighty-seven (33.0%) patients died due to all-cause mortality. The preoperative cholinesterase level was 5910 ± 1700 U/L. We divided cholinesterase concentrations into three groups. Table 1 lists the demographic and clinical characteristics of all 2387 patients and includes comorbidities, factors associated with injuries, and treatment.

**Table 1:** The demographic and clinical characteristics (*N* = 2387)

**Cholinesterase group**	** < 5000 U/L**	** ≥ 5000 U/L, < 7000 U/L**	** ≥ 7000 U/L**	***P* value**	*P* value*
**No. of patients**	732	1103	552		
**Age (year)**	82.24 ± 6.39	79.23 ± 6.63	76.51 ± 6.23	< 0.001	< 0.001
**Gender**				< 0.001	-
Male	285 (38.93%)	372 (33.73%)	124 (22.46%)		
Female	447 (61.07%)	731 (66.27%)	428 (77.54%)		
**Occupation**				0.296	-
Retirement	415 (56.69%)	663 (60.11%)	308 (55.80%)		
Farmer	183 (25.00%)	257 (23.30%)	132 (23.91%)		
Other	134 (18.31%)	183 (16.59%)	112 (20.29%)		
**History of allergy**				0.676	-
No	702 (95.90%)	1055 (95.65%)	533 (96.56%)		
Yes	30 (4.10%)	48 (4.35%)	19 (3.44%)		
**Injury mechanism**				0.025	-
Falling	702 (95.90%)	1070 (97.01%)	535 (96.92%)		
Accident	18 (2.46%)	29 (2.63%)	15 (2.72%)		
Other	12 (1.64%)	4 (0.36%)	2 (0.36%)		
**Fracture classification**				< 0.001	-
Intertrochanteric fracture	593 (81.01%)	793 (71.89%)	319 (57.79%)		
Femoral neck fracture	119 (16.26%)	282 (25.57%)	221 (40.04%)		
Subtrochanteric fracture	20 (2.73%)	28 (2.54%)	12 (2.17%)		
**aCCI**				< 0.001	-
2	15 (2.05%)	43 (3.90%)	46 (8.33%)		
3	75 (10.25%)	241 (21.85%)	148 (26.81%)		
4	319 (43.58%)	434 (39.35%)	214 (38.77%)		
5	214 (29.23%)	268 (24.30%)	101 (18.30%)		
6	83 (11.34%)	94 (8.52%)	30 (5.43%)		
7	24 (3.28%)	17 (1.54%)	12 (2.17%)		
8	2 (0.27%)	5 (0.45%)	1 (0.18%)		
9	0 (0.00%)	1 (0.09%)	0 (0.00%)		
**Hypertension**				< 0.001	-
No	430 (58.74%)	541 (49.05%)	250 (45.29%)		
Yes	302 (41.26%)	562 (50.95%)	302 (54.71%)		
**Diabetes**				< 0.001	-
No	629 (85.93%)	872 (79.06%)	408 (73.91%)		
Yes	103 (14.07%)	231 (20.94%)	144 (26.09%)		
**CHD**				0.034	-
No	317 (43.31%)	545 (49.41%)	265 (48.01%)		
Yes	415 (56.69%)	558 (50.59%)	287 (51.99%)		
**Arrhythmia**				< 0.001	-
No	433 (59.15%)	773 (70.08%)	393 (71.20%)		
Yes	299 (40.85%)	330 (29.92%)	159 (28.80%)		
**Hemorrhagic stroke**				0.069	-
No	709 (96.86%)	1083 (98.19%)	544 (98.55%)		
Yes	23 (3.14%)	20 (1.81%)	8 (1.45%)		
**Ischemic stroke**				0.508	-
No	510 (69.67%)	784 (71.08%)	401 (72.64%)		
Yes	222 (30.33%)	319 (28.92%)	151 (27.36%)		
**Cancer**				0.042	-
No	701 (95.77%)	1074 (97.37%)	541 (98.01%)		
Yes	31 (4.23%)	29 (2.63%)	11 (1.99%)		
**Associated injuries**				0.035	-
No	666 (90.98%)	1032 (93.56%)	521 (94.38%)		
Yes	66 (9.02%)	71 (6.44%)	31 (5.62%)		
**Dementia**				0.001	-
No	686 (93.72%)	1065 (96.55%)	538 (97.46%)		
Yes	46 (6.28%)	38 (3.45%)	14 (2.54%)		
**COPD**				0.027	-
No	677 (92.49%)	1028 (93.20%)	530 (96.01%)		
Yes	55 (7.51%)	75 (6.80%)	22 (3.99%)		
**Virus hepatitis**				0.045	-
No	699 (95.49%)	1071 (97.10%)	540 (97.83%)		
Yes	33 (4.51%)	32 (2.90%)	12 (2.17%)		
**Gastritis**				0.617	-
No	717 (97.95%)	1087 (98.55%)	543 (98.37%)		
Yes	15 (2.05%)	16 (1.45%)	9 (1.63%)		
**Treatment strategy**				< 0.001	-
Conservation	102 (13.93%)	72 (6.53%)	21 (3.80%)		
ORIF	514 (70.22%)	756 (68.54%)	317 (57.43%)		
HA	115 (15.71%)	262 (23.75%)	194 (35.14%)		
THA	1 (0.14%)	13 (1.18%)	20 (3.62%)		
**Time to admission (h)**	122.38 ± 334.64	65.26 ± 211.11	52.07 ± 209.43	< 0.001	< 0.001
**Time to operation (d)**	4.51 ± 2.93	4.19 ± 2.33	4.23 ± 2.43	0.035	0.091
**Operation time (mins)**	97.84 ± 39.46	92.45 ± 36.96	95.05 ± 34.84	0.016	0.012
**Blood loss (mL)**	261.97 ± 168.35	242.74 ± 153.89	229.21 ± 152.63	0.002	0.098
**Infusion (mL)**	1543.74 ± 417.19	1558.61 ± 378.33	1611.81 ± 370.13	0.009	< 0.001
**Transfusion (U)**	1.58 ± 1.29	1.10 ± 1.26	0.70 ± 1.09	< 0.001	< 0.001
**Stay in hospital (d)**	9.17 ± 3.73	8.81 ± 3.83	8.56 ± 2.98	0.01	0.012
**Follow-up (months)**	32.68 ± 19.62	39.94 ± 18.37	39.65 ± 15.98	< 0.001	< 0.001
**Mortality**				< 0.001	-
Survival	354 (48.36%)	801 (72.62%)	445 (80.62%)		
Dead	378 (51.64%)	302 (27.38%)	107 (19.38%)		

*CHD* coronary heart disease, *COPD* chronic obstructive pulmonary disease, *aCCI* age-adjusted Charlson comorbidity index

Mean + SD/*N*(%)*.* *For continuous variables, we used the Kruskal-Wallis rank-sum test, and Fisher’s exact probability test for count variables with a theoretical number < 10

### Univariate analysis of the association between variates and mortality

We performed univariate analysis to identify potential confounding factors and the relationship between variables and mortality (Table 2). According to the criteria of *P* < 0.1, the following variables were considered in the multivariate Cox regression: age, gender, injury mechanism, fracture classification, aCCI, CHD, arrhythmia, ischemic stroke, cancer, dementia, COPD, virus hepatitis, time to operation, treatment strategy, operation time, infusion, transfusion, and stay in hospital.

**Table 2:** Effects of factors on mortality measured by univariate analysis (*N* = 2387)

	**Statistics**	**HR (95% CI)**	***P* value**
**Age (year)**	79.52 ± 6.80	1.08 (1.07, 1.09)	< 0.0001
**Gender**			
Male	781 (32.72%)	1	
Female	1606 (67.28%)	0.73 (0.63, 0.84)	< 0.0001
**Occupation**			
Retirement	1386 (58.06%)	1	
Farmer	572 (23.96%)	0.93 (0.78, 1.10)	0.3965
Other	429 (17.97%)	0.88 (0.72, 1.06)	0.1775
**History of allergy**			
No	2290 (95.94%)	1	
Yes	97 (4.06%)	0.93 (0.64, 1.35)	0.7158
**Injury mechanism**			
Falling	2307 (96.65%)	1	
Accident	62 (2.60%)	0.24 (0.11, 0.54)	0.0006
Other	18 (0.75%)	1.60 (0.83, 3.09)	0.1593
**Fracture classification**			
Intertrochanteric fracture	1705 (71.43%)	1	
Femoral neck fracture	622 (26.06%)	0.86 (0.72, 1.03)	0.092
Subtrochanteric fracture	60 (2.51%)	0.69 (0.43, 1.12)	0.1348
**Stay in hospital (d)**	8.86 ± 3.62	1.03 (1.01, 1.05)	0.0013
**aCCI**			
2	104 (4.36%)	1	
3	464 (19.44%)	2.81 (1.22, 6.46)	0.0148
4	967 (40.51%)	6.77 (3.02, 15.19)	< 0.0001
5	583 (24.42%)	9.31 (4.14, 20.92)	< 0.0001
6	207 (8.67%)	11.78 (5.17, 26.84)	< 0.0001
7	53 (2.22%)	15.71 (6.54, 37.77)	< 0.0001
8	8 (0.34%)	29.32 (9.45, 91.03)	< 0.0001
9	1 (0.04%)	31.74 (3.82, 263.93)	0.0014
**Hypertension**			
No	1221 (51.15%)	1	
Yes	1166 (48.85%)	1.12 (0.97, 1.29)	0.1133
**Diabetes**			
No	1909 (79.97%)	1	
Yes	478 (20.03%)	0.99 (0.83, 1.18)	0.8672
**CHD**			
No	1127 (47.21%)	1	
Yes	1260 (52.79%)	1.35 (1.17, 1.55)	< 0.0001
**Arrhythmia**			
No	1599 (66.99%)	1	
Yes	788 (33.01%)	1.31 (1.14, 1.51)	0.0002
**Hemorrhagic stroke**			
No	2336 (97.86%)	1	
Yes	51 (2.14%)	1.11 (0.70, 1.77)	0.6561
**Ischemic stroke**			
No	1695 (71.01%)	1	
Yes	692 (28.99%)	1.44 (1.24, 1.67)	< 0.0001
**Cancer**			
No	2316 (97.03%)	1	
Yes	71 (2.97%)	1.79 (1.29, 2.50)	0.0005
**Associated injuries**			
No	2219 (92.96%)	1	
Yes	168 (7.04%)	0.93 (0.70, 1.24)	0.6313
**Dementia**			
No	2289 (95.89%)	1	
Yes	98 (4.11%)	2.81 (2.17, 3.65)	< 0.0001
**COPD**			
No	2235 (93.63%)	1	
Yes	152 (6.37%)	1.54 (1.20, 1.97)	0.0006
**Virus hepatitis**			
No	2310 (96.77%)	1	
Yes	77 (3.23%)	1.46 (1.04, 2.06)	0.0274
**Gastritis**			
No	2347 (98.32%)	1	
Yes	40 (1.68%)	0.97 (0.57, 1.65)	0.9122
**Time to admission (h)**	79.73 ± 256.62	1.00 (1.00, 1.00)	0.1107
**Time to operation (d)**	4.29 ± 2.54	1.03 (1.00, 1.06)	0.0427
**Treatment strategy**			
Conservation	195 (8.17%)	1	
ORIF	1587 (66.49%)	0.30 (0.25, 0.36)	< 0.0001
HA	571 (23.92%)	0.32 (0.26, 0.41)	< 0.0001
THA	34 (1.42%)	0.06 (0.02, 0.25)	< 0.0001
**Operation time (mins)**	94.63 ± 37.26	1.00 (1.00, 1.00)	0.0849
**Blood loss (mL)**	245.09 ± 158.31	1.00 (1.00, 1.00)	0.5711
**Infusion (mL)**	1567.08 ± 388.57	1.00 (1.00, 1.00)	0.0003
**Transfusion (U)**	1.14 ± 1.27	1.06 (1.00, 1.13)	0.0344
**Cholinesterase (U/L)**	5910 ± 1700	0.74 (0.70, 0.77)	< 0.0001

*CHD* coronary heart disease, *COPD* chronic obstructive pulmonary disease, *aCCI* age-adjusted Charlson comorbidity index

### Multivariate analysis between preoperative cholinesterase and mortality

We used three models (Table 3) to correlate preoperative cholinesterase levels and mortality. When cholinesterase concentration was a continuous variable, a linear regression was observed. The fully adjusted model showed a mortality risk decrease of 17% (HR = 0.83, 95%CI: 0.78–0.88), *P* < 0.0001) when cholinesterase concentration increased by 1000 U/L after controlling for confounding factors. When cholinesterase concentration was used as a categorical variable, we found statistically significant differences in the cholinesterase levels among the three models (*P* < 0.0001). In addition, the *P* for trend also showed a linear correlation in these three models (*P* < 0.0001).

**Table 3:** Univariate and multivariate results by cox regression (*N* = 2387)

**Exposure**	**Non-adjusted model**	**Minimally adjusted model**	**Fully adjusted model**
**Cholinesterase**	0.74 (0.70, 0.77) < 0.0001	0.80 (0.76, 0.84) < 0.0001	0.83 (0.78, 0.88) < 0.0001
**Cholinesterase** **group**			
** < 5000** **U/L**	Ref	Ref	Ref
** ≥ 5000** **U/L, < 7000** **U/L**	0.44 (0.38, 0.51) < 0.0001	0.52 (0.44, 0.60) < 0.0001	0.57 (0.48, 0.68) < 0.0001
** ≥ 7000** **U/L**	0.31 (0.25, 0.39) < 0.0001	0.46 (0.37, 0.57) < 0.0001	0.55 (0.42, 0.70) < 0.0001
***P*** **for** **trend**	< 0.0001	< 0.0001	< 0.0001

Data in table: HR (95%CI) *P* value

Outcome variable: mortality

Exposed variables: preoperative cholinesterase

Minimally adjusted adjust for: age; gender

Fully adjusted model adjust for: age; gender; injury mechanism; fracture classification; aCCI; CHD; arrhythmia; ischemic stroke; cancer; dementia; COPD; virus hepatitis; time to operation; treatment strategy; operation time; infusion; transfusion; stay in hospital

However, we found that the changing interval was slow in the subgroup with a cholinesterase level ≥ 7000 U/L (Table 3). This instability indicated the possibility of a nonlinear correlation.

### Curve fitting and analysis of threshold effect

**Fig. 1:**
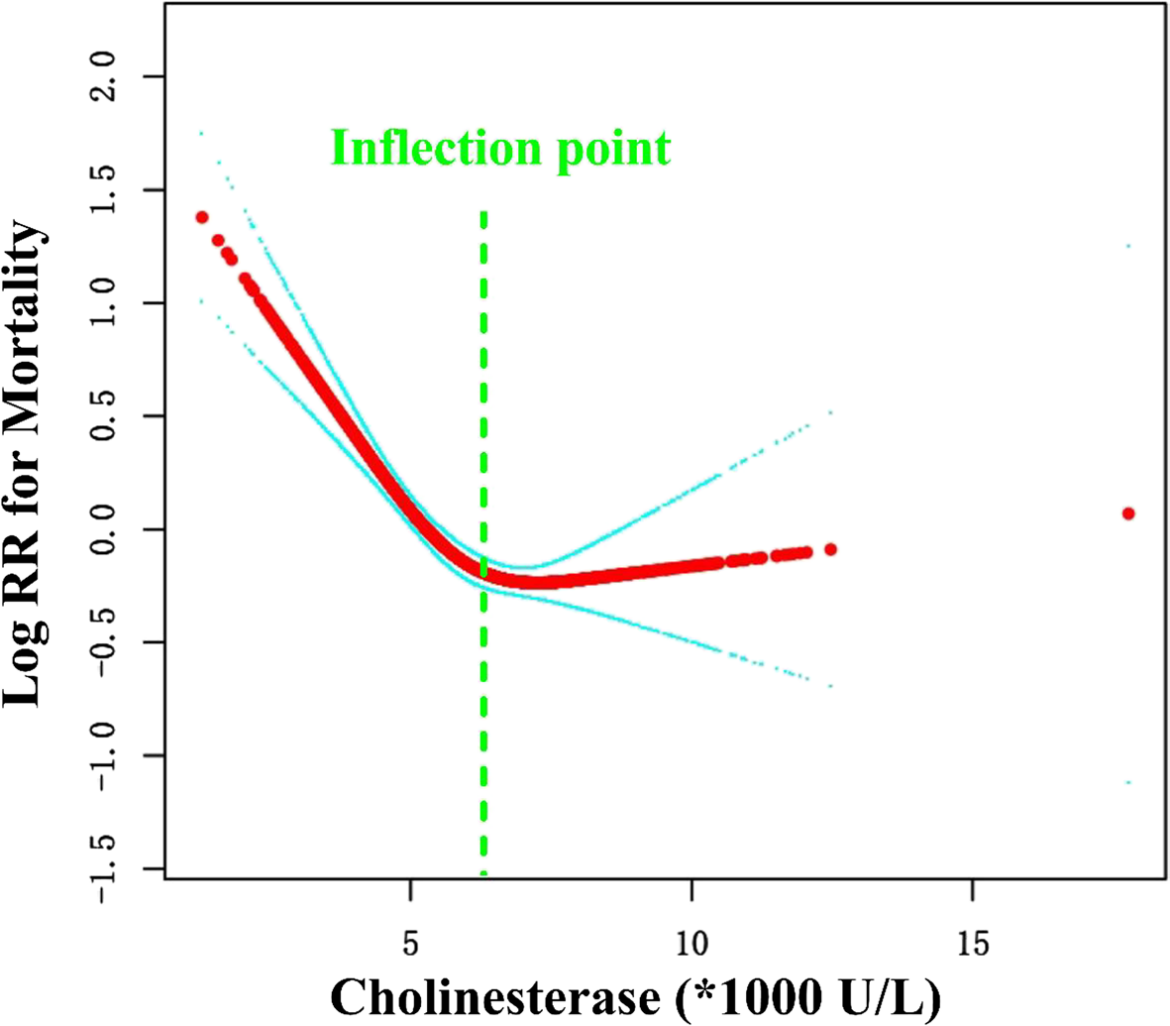
Curve fitting between preoperative cholinesterase and mortality. Adjusted for age; gender; injury mechanism; fracture classification; aCCI; CHD; arrhythmia; ischemic stroke; cancer; dementia; COPD; virus hepatitis; time to operation; treatment strategy; operation time; infusion; transfusion; and stay in hospital

**Fig. 2:**
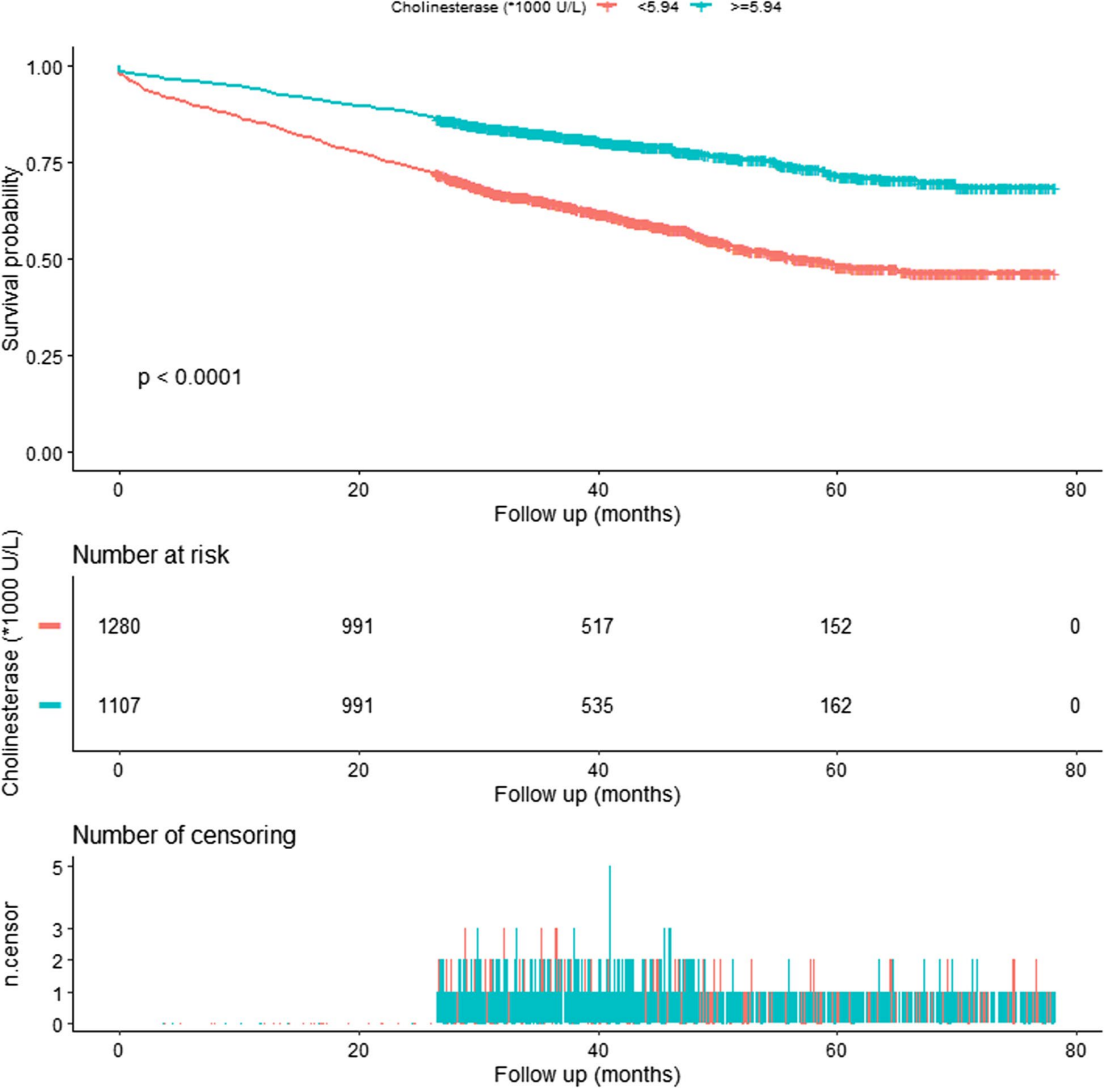
The Kaplan-Meier survival curve

As shown in Fig. 1, there was a curved association between preoperative cholinesterase level and mortality after adjusting for confounding factors. We compared two fitting models to explain this association (Table 4). Interestingly, we observed an inflection point in the saturation effect. When preoperative cholinesterase level < 5940 U/L, the mortality decreased by 28% for every 1000 U/L increase in cholinesterase (HR = 0.72, 95%CI: 0.66–0.79, *P* < 0.0001). When cholinesterase was > 5940 U/L, the mortality was no longer decreased with the rise of cholinesterase (HR = 1.01, 95%CI: 0.91–1.11, *P* = 0.9157). The Kaplan–Meier survival curve is shown in Fig. 2.

**Table 4:** Nonlinearity of preoperative cholinesterase (*1000 U/L) versus mortality (*N* = 2387)

Outcome	HR (95%CI) *P* value
Fitting model by stand linear regression	0.83 (0.78, 0.88) < 0.0001
Fitting model by two-piecewise linear regression	
Inflection point	5940
< 5940	0.72 (0.66, 0.79) < 0.0001
> 5940	1.01 (0.91, 1.11) 0.9157
*P* for log-likelihood ratio test	< 0.001

Adjust for: age; gender; injury mechanism; fracture classification; aCCI; CHD; arrhythmia; ischemic stroke; cancer; dementia; COPD; virus hepatitis; time to operation; treatment strategy; operation time; infusion; transfusion; and stay in hospital

### Propensity score matching (PSM)

**Fig. 3:**
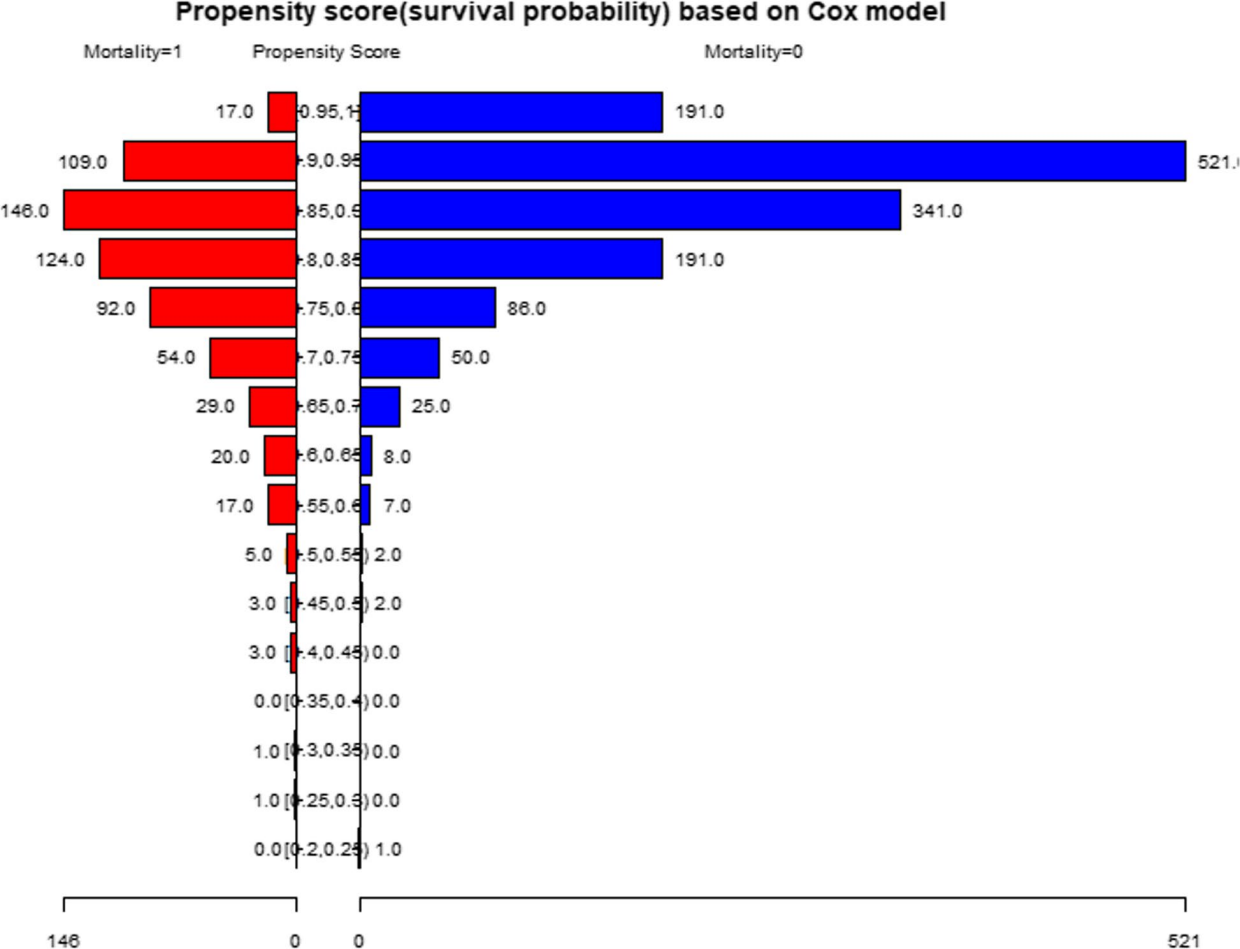
The PSM of two groups under propensity score based on Cox model

To test the robustness of our results, we performed sensitivity analysis using PSM, as shown in Fig. 3 and Tables 5, 6 and 7. There were 1264 patients (53.00%) successfully matched (Fig. 1; Table 5). Age and aCCI treatment did not match between the two groups (Table 6). We found that the results were stable in the multivariate Cox regression results under the PSM and PSM-adjusted models (Table 7).

**Table 5:** Propensity score parameter list

**The variables used in calculating the propensity score**	**Age; gender; injury mechanism; fracture classification; aCCI; CHD; arrhythmia; ischemic stroke; cancer; dementia; COPD; virus hepatitis; time to operation; treatment strategy; operation time; infusion; transfusion; stay in hospital**
Propensity score algorithm	Cox regression model
C-statistical	0.71
Matching method	Greedy matching within specified caliper distances
Metric distances	0.05
Matching ratio	1:1
Use of replacement	With replacement
Matching sample size	No. of mortality = 1: 632 cases No. of mortality = 0: 632 cases Total 1264 cases

**Table 6:** The balance test of PSM (*N* = 1264)

**Variables**	**Mortality: survival (*N* = 632)**	**Mortality: dead (*N* = 632)**	**Standardized diff**	***P* value**
**Age (year)**	83.38 ± 4.42	81.99 ± 6.35	0.2544	< 0.0001*
**Gender**			0.0559	0.3503
Male	223 (35.3)	240 (38)		
Female	409 (64.7)	392 (62)		
**Injury mechanism**				0.4958
Falling	618 (97.8)	620 (98.1)	0.0223	
Accident	10 (1.6)	6 (0.9)	0.0566	
Other	4 (0.6)	6 (0.9)	0.0357	
**Fracture classification**				0.0598
Intertrochanteric fracture	445 (70.4)	473 (74.8)	0.0995	
Femoral neck fracture	181 (28.6)	148 (23.4)	0.1192	
Subtrochanteric fracture	6 (0.9)	11 (1.7)	0.0687	
**aCCI**				< 0.0001*
2	0 (0)	6 (0.9)	0.1385	
3	19 (3)	67 (10.6)	0.3051	
4	278 (44)	273 (43.2)	0.016	
5	235 (37.2)	191 (30.2)	0.1477	
6	82 (13)	73 (11.6)	0.0434	
7	16 (2.5)	18 (2.8)	0.0196	
8	2 (0.3)	4 (0.6)	0.0461	
**CHD**			0.0032	1
No	282 (44.6)	283 (44.8)		
Yes	350 (55.4)	349 (55.2)		
**Arrhythmia**			0.0265	0.6803
No	405 (64.1)	413 (65.3)		
Yes	227 (35.9)	219 (34.7)		
**Ischemic stroke**			0.0169	0.8102
No	424 (67.1)	429 (67.9)		
Yes	208 (32.9)	203 (32.1)		
**Cancer**			0.0563	0.3911
No	610 (96.5)	603 (95.4)		
Yes	22 (3.5)	29 (4.6)		
**Dementia**			0.0957	0.1146
No	603 (95.4)	589 (93.2)		
Yes	29 (4.6)	43 (6.8)		
**COPD**			0.0186	0.826
No	589 (93.2)	586 (92.7)		
Yes	43 (6.8)	46 (7.3)		
**Virus hepatitis**			0.0079	1
No	606 (95.9)	605 (95.7)		
Yes	26 (4.1)	27 (4.3)		
**Treatment strategy**				0.086
ORIF	441 (69.8)	476 (75.3)	0.1243	
HA	189 (29.9)	154 (24.4)	0.1248	
THA	2 (0.3)	2 (0.3)	0	
**Transfusion (U)**				0.7442
0	289 (45.7)	276 (43.7)	0.0414	
1	2 (0.3)	2 (0.3)	0	
2	297 (47)	307 (48.6)	0.0317	
3	0 (0)	2 (0.3)	0.0797	
4	41 (6.5)	43 (6.8)	0.0127	
6	3 (0.5)	2 (0.3)	0.0252	
**Time to operation (d)**	4.45 ± 2.54	4.51 ± 2.83	0.0241	0.6681
**Operation time (mins)**	91.46 ± 34.62	92.23 ± 33.66	0.0227	0.6865
**Infusion (mL)**	1506.57 ± 347.69	1512.25 ± 372.73	0.0158	0.7794
**Stay in hospital (d)**	9.02 ± 3.64	9.05 ± 3.45	0.0094	0.8678

^*^Variables were not successfully matched

**Table 7:** Multivariate results by Cox regression (*N* = 1264)

**Outcome**	**Fully adjusted model**	**PSM model**	**PSM-adjusted model**
**Fitting model by stand linear regression**	0.83 (0.78, 0.88) < 0.0001	0.89 (0.84, 0.94) < 0.0001	0.88 (0.83, 0.92) < 0.0001
**Fitting model by two-piecewise linear regression**			
**Inflection point**	5940	5840	5840
** < Inflection point**	0.72 (0.66, 0.79) < 0.0001	0.76 (0.70, 0.83) < 0.0001	0.74 (0.68, 0.81) < 0.0001
** > Inflection point**	1.01 (0.91, 1.11) 0.9157	1.07 (0.98, 1.17) 0.1302	1.06 (0.97, 1.16) 0.2104
***P* for log-likelihood ratio test**	< 0.001	< 0.001	< 0.001

Data in table: HR (95% CI) *P* value

Outcome variable: mortality

Exposed variables: cholinesterase

Adjust variables in PSM-adjusted model: age, aCCI

## Discussion

The serum cholinesterase indicates liver function (Meng et al. 2013; Kaufman 1954; Tan et al. 2019) and is closely associated with the synthesis of albumin in the liver (Levine and Hoyt 1950) and is a well-known marker of liver dysfunction. As a physiologic indicator, cholinesterase has its normal range of 5000–12,000 U/L. A high level of cholinesterase means fine function and better status in liver, which was associated with low mortality rate. In this study, we found a curved association between preoperative cholinesterase level and mortality, and a concentration of 5940 U/L was an inflection point in the saturation effect. When preoperative cholinesterase level < 5940 U/L, the mortality decreased by 28% for every 1000 U/L increase in cholinesterase (HR = 0.72). When cholinesterase was > 5940 U/L, the mortality was no longer decreased with the rise of cholinesterase (HR = 1.01). Thus, a preoperative cholinesterase of 5940 U/L was a useful indicator to predict mortality in the clinical setting.

Several studies have revealed associations between serum cholinesterase and acute pancreatitis (Wei et al. 2022), colorectal cancer (Takano et al. 2022), non-small-cell lung cancer (Ran et al. 2022), and Alzheimer’s disease (Shahid Nadeem et al. 2022; Sharma et al. 2020; Sharma 2019). There was no evidence of hip or other fractures. To the best of our knowledge, this is the first study to investigate the relationship between cholinesterase levels and mortality in geriatric patients with hip fractures. Serum cholinesterase is a common indicator of liver function (Meng et al. 2013; Kaufman 1954; Tan et al. 2019) with good sensitivity and specificity (Abbas and Abbas 2017; Ramachandran et al. 2014). Serum cholinesterase levels may be decreased due to reduced cholinesterase synthesis in those with liver dysfunction. This contrasts with other serum enzymes associated with the clinical assessment of liver function, whose activities increase due to enhanced release from cellular sources following cellular membrane damage (Meng et al. 2013). Some studies have reported an association between liver function and mortality after hip fractures. Hundersmarck et al. concluded that worsening liver function is associated with increased mortality (Hundersmarck et al. 2021). Montomoli et al. reported that liver disease patients had increased 30-day mortality and 1-year mortality following hip fractures compared to patients without liver disease (Montomoli et al. 2018). In addition, compared with the general group, the cirrhosis group had two to three times higher mortality rates at 3 months and 1 year (Chang et al. 2020). Thus, low liver function is associated with a poor prognosis. It is reasonable to assume that the serum cholinesterase level, as a reflection of liver function, is related to prognosis.

In this study, we established an association using curve fitting and found a saturation point, thereby identifying a meaningful predictive point. When cholinesterase concentration was < 5940 U/L, the mortality was higher than in patients with cholinesterase concentration > 5940 U/L. In fact, serum cholinesterase level is also an independent predictor of all-cause mortality in the general community-based population, as reported by Saegusa et al. (Saegusa et al. 2022) Cholinesterase concentration is closely related to liver synthetic function (Ramachandran et al. 2014), such as albumin, prealbumin, and prothrombin time, which are markers of synthetic liver function. Our study shows that cholinesterase levels are also associated with mid-term mortality in patients with hip fractures. Thus, serum cholinesterase was an independent predictor of mortality, according to the cut-off of 5940 U/L. Our findings provide a new perspective on the predictive role of preoperative cholinesterase, and it calls for a study on the pathophysiology and significant changes in liver function after hip fractures in the elderly.

To explore possible confounders in the study, we identified the factors affecting prognosis and cholinesterase levels. The main factors affecting prognosis are shown in Table 2. Age (Xu et al. 2019), gender (Guzon-Illescas et al. 2019), fracture classification (Xu et al. 2019), aCCI (Abeygunasekara et al. 2020), CHD (Kilci et al. 2016), arrhythmia (Frenkel et al. 2021; Abu-Assi et al. 2020), cancer (Hemelrijck et al. 2013), dementia (Hou et al. 2021), COPD (Barcelo et al. 2021), time to operation (Kristiansson et al. 2020), operation (Tang et al. 2012), transfusion (Greenhalgh et al. 2021), and stay in hospital (Hommel et al. 2008) were reported as risk factors in previous studies. In addition, we also found associations between mortality and injury mechanism, ischemic stroke, operation time, and infusion in univariate analysis. As for the factors influencing cholinesterase concentration, we included virus hepatitis because of its relationship with liver function (Watson and Hoffbauer 1947). Therefore, several possible confounders were considered in this study.

In our study, the longest follow-up period was 84.19 months (mean, 37.64 months). Furthermore, we included patients who were admitted no later than September 2019 to avoid complications from COVID-19 (Levitt et al. 2022; Zhong et al. 2021) and for a follow-up period of at least 2 years. To assess the relationship between cholinesterase concentration and mortality, we performed linear regression to the adjusted model; we had also considered factors that were included in earlier studies (Takagi et al. 2022; Pan et al. 2022; Chen et al. 2022; Bicen et al. 2021; Hjelholt et al. 2022; Giovanni et al. 2021; Demirel and Sahin 2021; Chiang et al. 2021; Sayed-Noor et al. 2021; Gatot et al. 2021; Liow et al. 2021). We adjusted the factor of *P* < 0.1 in the univariate analysis, and we comprehensively considered variables that needed adjustment. Specifically, we use a sensitivity analysis of the trend test in the linear model. In addition, because of the inconsistent HR interval of the model, we considered the curve association and found a clinical saturation effect and inflection point. Curve fitting was more suitable than linear fitting for explaining the association between preoperative cholinesterase and mortality. Specifically, we used PSM analysis in the nonlinear model and found that the nonlinear association was very stable.

Our study had some limitations. Firstly, due to the cohort study design, those lost to follow-up comprised 17.3%, which was inevitable. To obtain a prognosis, we tried to contact patients who did not answer three times. Secondly, all patients in this study were from China; thus, our findings have regional and ethnic restrictions, and inflection points should be defined for other populations. Thirdly, 77 (3.23%) of patients suffered the virus hepatitis but could not be divided the level of liver dysfunction and cirrhosis because short of the corresponding data.

## Conclusion

In summary, preoperative cholinesterase levels were nonlinearly associated with mortality in elderly hip fractures, and cholinesterase was a risk indicator of all-cause mortality.

## Data Availability

The data was implemented by Xi’an Honghui Hospital. According to relevant regulations, the data could not be shared, but could request from correspondence author.
